# *Aedes aegypti* Control Strategies in Brazil: Incorporation of New Technologies to Overcome the Persistence of Dengue Epidemics

**DOI:** 10.3390/insects6020576

**Published:** 2015-06-11

**Authors:** Helena R. C. Araújo, Danilo O. Carvalho, Rafaella S. Ioshino, André L. Costa-da-Silva, Margareth L. Capurro

**Affiliations:** Departamento de Parasitologia, Instituto de Ciências Biomédicas, Universidade de São Paulo, São Paulo SP 05508-000, Brazil; E-Mails: helenarca@gmail.com (H.R.C.A.); cdanilo@usp.br (D.O.C.); myxelia@gmail.com (R.S.I.); alcosta@icb.usp.br (A.L.C.S.)

**Keywords:** vector control, Brazil, PNCD, integrated mosquito management

## Abstract

Dengue is considered to be the most important mosquito-borne viral disease in the world. The *Aedes aegypti* mosquito, its vector, is highly anthropophilic and is very well adapted to urban environments. Although several vaccine candidates are in advanced stages of development no licensed dengue vaccine is yet available. As a result, controlling the spread of dengue still requires that mosquitoes be targeted directly. We review the current methods of dengue vector control focusing on recent technical advances. We first examine the history of Brazil’s National Dengue Control Plan in effect since 2002, and we describe its establishment and operation. With the persistent recurrence of dengue epidemics, current strategies should be reassessed to bring to the forefront a discussion of the possible implementation of new technologies in Brazil’s mosquito control program.

## 1. Introduction

Mosquito-borne diseases are among the leading causes of mortality and morbidity in humans. Globalization of travel and trade, unplanned urbanization and environmental changes have all had a significant impact on disease transmission in recent years. Some arboviruses, such as dengue, chikungunya and West Nile virus, are emerging in countries where they have previously been unknown [1]. Another example of a mosquito-borne disease is the Zika fever (transmitted by a dengue-related flavivirus), which has not previously been thought to be an important human arboviral pathogen. The epidemic capacity of the Zika virus was revealed in an outbreak in Micronesia in 2007 and affected approximately 5000 people [2]. Dengue has stood out among the reemerging diseases, and it is considered to be the most important viral disease transmitted by arthropods. In 2013, it was estimated that approximately 390 million people experienced dengue virus infections per year, of which 96 million cases had some kind of apparent manifestation [3]. The Americas are not an exception to this trend, and the incidence of dengue in the Americas has increased 30-fold in the last 50 years; between 2008 and 2012, more than 1.2 million cases of dengue were identified each year. Furthermore, 2013 had the highest burden of the disease ever registered with the largest epidemic in the history of the Americas. A total of 2.3 million cases were reported [4] and Brazil was responsible for 1,452,489 of those cases according to the Ministry of Health [5].

Dengue was first registered in Brazil in 1981 in Boa Vista in the north of the country; this outbreak of serotypes DENV-1 and DENV-4 was rapidly suppressed. In 1986, dengue acquired epidemiological importance when an epidemic broke out in the state of Rio de Janeiro with Serotype 1. The virus spread throughout seven states with periodic outbreaks until 1990 when Serotype 2 was also introduced in the Rio de Janeiro state. Since 1994, there has been a rapid dispersal of the vector over a large area, which led to a viral circulation in more states and municipalities and caused a rapid increase in the extent of the disease [6]. In Brazil, DENV-3 was first isolated in 2000 from the state of Rio de Janeiro. Later there were large DENV-3 epidemics in Rio de Janeiro in 2001 and 2002 [7]. DENV-4 reemerged in Brazil in 2010, 28 years after it had last been detected in the country; the site of the reemergence was Roraima state in northern Brazil [8].

The first vector control effort in Brazil was focused on the control of the yellow fever virus through a partnership with the Rockefeller Foundation and the Brazilian National Department of Public Health. Actions aimed at vector control using insecticides started in 1947. The advent of DDT made it possible to imagine the notion of mosquito eradication not only on a nationwide scale, but on a continent-wide scale. This mindset led to the Pan American Health Organization (PAHO) and the World Health Organization (WHO) forming a new group to fight mosquitoes: the Eradication Program of *Ae. aegypti* in the Western Hemisphere. Thanks to these programs, *Ae. aegypti* was considered to be eradicated from Brazil by the end of the 1950s [6,9,10].

In 1956, the National Department of Rural Endemic Diseases (DNERu) was created to control yellow fever and malaria. After 10 years, *Ae. aegypti* was reintroduced and the responsibilities of DNERu were delegated to the Superintendence of Public Health Campaigns (SUCAM) through the National Program of Yellow Fever and Dengue Control. This campaign was disbanded in 1973, and the vector was, once more, considered to be eradicated from the country. However, in 1976 it was reintroduced due to failures in epidemiological surveillance and social and environmental changes resulting from rapid urbanization [6]. The deterioration of the eradication program over time was due mainly to the development of mosquito resistance to DDT and other organochlorine insecticides, the high costs of materials and wages, insufficient community participation or support from the health sector, and an unwillingness on the part of some governments to join in simultaneous programs [10].

The Brazilian National Health Foundation (FUNASA) was placed in charge of the vector and disease control in 1990. Its first strategy was to develop the Eradication Plan of *Ae. aegypti* (PEAa). However, this plan failed because of the uncoordinated actions of each municipality and inconsistencies in the implementation of actions to combat the vector. FUNASA then gave up on eradicating the mosquito and focused instead on controlling the vector through the Intensification Plan of Dengue Control Actions (PIACD) in 2001. Some changes were enacted in 2002, and the final National Plan for Dengue Control (PNCD) was established [6]. The Brazilian authorities created this compendium by compiling different strategies to control dengue disease in the national territory. The PNCD addresses important components needed to control dengue such as epidemiologic surveillance, vector control, patient care, integration with primary health care through public health agents, environmental sanitation activities, integrated actions of health education, communication and social mobilization, training of human resources, political legislation for social sustainability, and program evaluation [11].

The main objective of the PNCD is to reduce *Ae. aegypti* infestation and thereby reduce the incidence of dengue and its lethal hemorrhagic manifestations. The goals of the PNCD are (1) to reduce the building infestation index in all priority cities considered to less than 1%, (2) to reduce, by 50%, the number cases of 2003 compared with the number of cases in 2002 and to sustain an additional 25% decrement each year and (3) to reduce the lethality of dengue hemorrhagic fever to less than 1% [11,12].

The PNCD’s procedures for mosquito control in Pernambuco state include a bimonthly application of temephos (larvicide) inside of houses with detectable breeding sites, an annual campaign for source elimination (Dengue Day) and less-frequent applications of organophosphorus or pyrethroids in ultra-low volumes in the case of epidemics for adult mosquito elimination. In some cases, temephos can be replaced by chitin synthesis inhibitors [13].

Despite all of the strategies implemented in Brazil by the PNCD, investigations of *Ae. aegypti,* populations in 67 cities revealed selective resistance to temephos in 1999 and 2000; this resistance was mainly found in northeastern and southeastern Brazil [6]. In a study conducted in two cities in Pernambuco state in the northeastern part of the country, the designated procedures from the PNCD were followed but yielded no positive suppression of the vector. The Oswaldo Cruz Foundation (FIOCRUZ) decided to implement a massive trap intervention for a couple of years, which resulted in a population suppression of approximately 90% [13].

**Figure 1:**
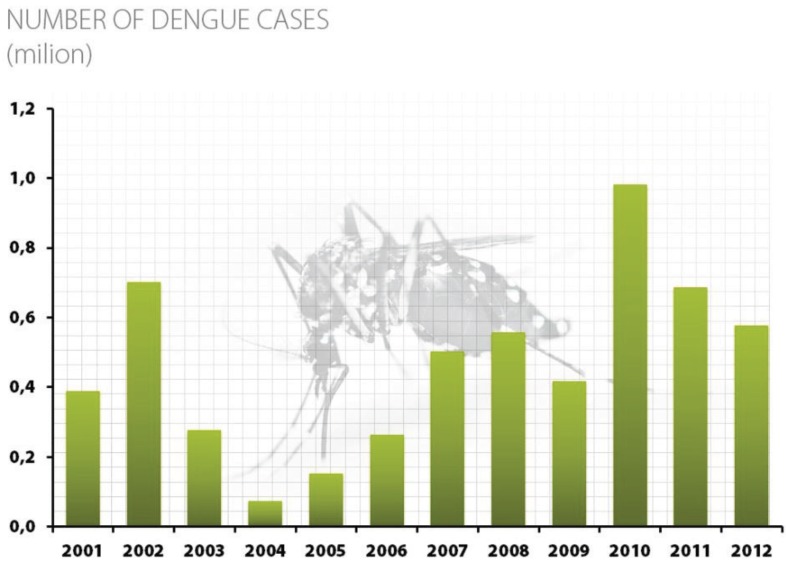
Number of dengue cases in Brazil. Source: Notifiable Diseases Information System [14].

When the PNCD was initiated in 2002, approximately 701,335 cases of dengue were reported in Brazil. In 2004, this number decreased to 72,552 cases. However, the number of cases then began to increase each year to 981,276 cases in 2010 (Figure 1). Ten years after the formation of the PNCD, approximately 4.5 million cases of dengue had been reported. These data were obtained from the Information System for Notifiable Diseases from the Brazilian Ministry of Health [14].

Whereas the PNCD has succeeded with PEAa, the vertically integrated features of the program to combat vector persist, and these features result in the poor coordination of prevention and control efforts with other health initiatives. Inefficiency results and goals have not been achieved as a result. Moreover, the occurrence of frequent outbreaks in recent years should serve to highlight that a new logic system is necessary for health service organizations: dengue control demands an entirely new set of new strategies and techniques to fight the vector and its disease [15].

Uncontrolled urbanization and the formation of megacities are generally accompanied by areas of poor sanitation and extreme poverty, which are optimal conditions for the establishment of vector breeding sites and dengue epidemics. Moreover, climate change has increased the domestic practice of storing water in containers, which is an ideal vector-breeding site. It is therefore critical that local characteristics and population habits be taken into consideration when choosing the most suitable program for mosquito control [16]. Below, we discuss several innovative tools that can be incorporated into vector control strategies.

## 2. Insecticides and Plant Extracts

Dengue virus transmission involves several factors such as the vector population density, the immunological profile of the human population, the circulating dengue serotypes, the climate and environmental conditions and the presence of other potential vector such as *Aedes albopictus* [17]. A commercial, large-scale and effective vaccine against all dengue serotypes does not exist; the most important strategy to suppress arbovirus disease transmission is vector control [18].

**Figure 2:**
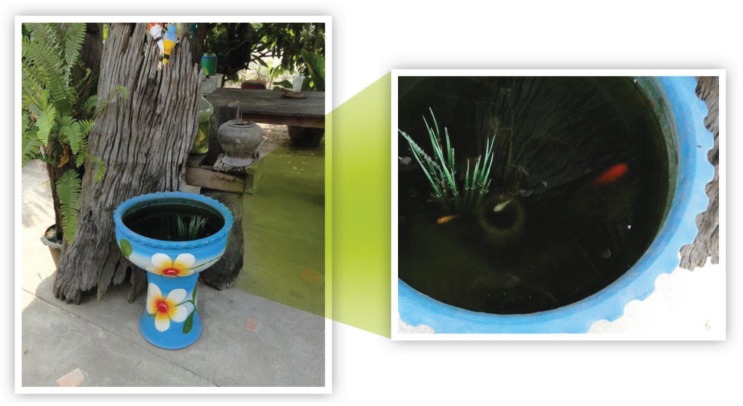
Larvivorous fish. Source: Pattamaporn Kittayapong and Phanthip Olanratmanee, Thailand.

According to the Brazilian national guidelines of the PNCD, vector control methods can be divided into mechanical, biological and chemical [19]. Mechanical control consists of removing potential breeding sites for mosquitoes such as tires and non-biodegradable plastic containers and covering water tanks in order to prevent oviposition by *Ae. aegypti* female mosquitoes. Biological control is designed to eliminate mostly mosquito larvae using biological agents. One option is to use *Bacillus thuringiensis israelensis* (Bti), a bacterium that, when ingested by mosquito larvae, synthesizes lethal endotoxins [19,20]. Pots with aquatic plants that are widely used in Southeast Asia to decorate houses and terraces provide fertile ground for mosquito breeding. Larvivorous fishes (*i.e*., those that feed upon larvae) are commonly used in such pots (Figure 2). This practice is common in India as an important component of a biological control strategy to reduce vector population and malaria transmission [21,22,23]. The use of larvivorous fish in intradomestic pots for mosquito control has proven to be an intelligent solution. Prohibiting the use of pots with aquatic plants is not effective since it contradicts habits that are deeply rooted in Asian culture.

Chemical control is performed using chemical compounds called insecticides, which can affect the larval or adult stages of mosquitoes. In the 1940s, mosquito control involved using synthetic insecticides, such as DDT, chlordane, benzene hexachloride and hexaethyl tetraphosphate [24]. Nowadays, the most common chemical compounds used in mosquito control include pyrethroids, organochloride and organophosphorus; these molecules interact with an insect’s nervous system [25]. However, because chemical control is part of an integrated control effort to combat the vector, its continuous use increases the selection of individuals that are resistant to those molecules. Furthermore, these same molecules can be toxic to other animals species or may contaminate the soil [24,26].

**Figure 3:**
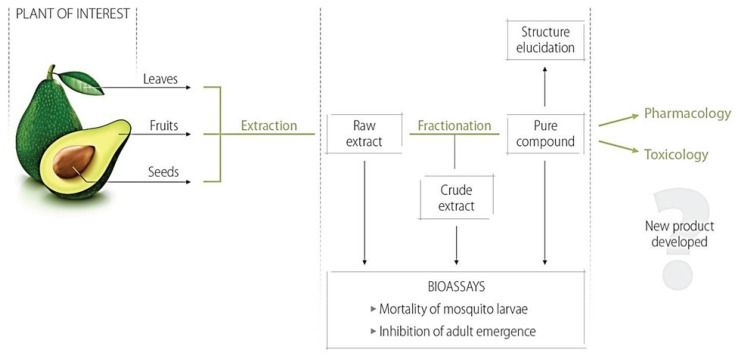
Strategies for obtaining active compounds from plants with larvicidal activity against mosquitoes.

Many researchers are developing new strategies to control and reduce the use of toxic products. One alternative is to use a botanical insecticide that is sustainable and less toxic than synthetic insecticides to combat *Ae. aegypti* mosquitoes [24,27,28]. Botanical insecticides can currently be synthesized from the extracts of leaves, seeds or fruits of many species of plants (Figure 3) such as *Apium graveolens*, *Persea americana*, *Cipadessa baccifera* and *Callistemon rigidus* [29,30,31,32]. It is possible to isolate chemical components from the extracts and perform larvicidal bioassays or tests using adult mosquitoes according to the Guidelines for Laboratory and Field Testing of Mosquito Larvicides published by the WHO [33,34,35].

The extracts can be used not only to produce insecticides but also to develop potential repellents. Citronella, for example, is a chemical compound present in aromatic plants (*Cymbopogon citratus* and *Eucalyptus citriodora*) and is used today for the production of mosquito repellents [36]. Kumar *et al.* (2014) also demonstrated that the oil from seeds of *Apium graveolens* was an effective repellent against *Ae. aegypti* [29].

Dias *et al.* (2014) performed a comprehensive review of the original research and described the bioactivity of essential oils against *Ae. aegypti* larvae. These authors showed that approximately 27% of the plants studied for their larvicidal activity were collected in Brazil [37].

There have been several studies that have identified natural compounds with insecticide or repellent characteristics [29,30,31,32]. In addition, Brazil encompasses significant biodiversity, and almost 19% of the world's flora grow in the country [38]. However, despite the number of studies conducted in Brazil it is important to maintain strict standards when searching for botanical insecticides: new classes of biomolecules should go through toxicological and other mandatory tests before being registered and released for large-scale production by the responsible agencies [39].

## 3. Paratransgenesis and *Wolbachia* Approaches

Some symbiotic bacteria have also been proposed to be used as an additional vector control method. The idea of exploiting microorganisms to combat mosquitoes is not new and was originally proposed more than 20 years ago. This strategy is also known as paratransgenesis but only when it involves the manipulation of symbionts for the production of anti-pathogen effector molecules [40,41]. Some important primary requirements for such symbionts are dominance, prevalence and stability within the insect body [40]. For example, a study using a green fluorescent protein-tagged *Asaia* strain showed that these bacteria colonized all mosquitoes. Furthermore, these bacteria massively colonized the larval gut and the male reproductive system. *Asaia* is also vertically transmitted, which corresponds to it quickly spreading throughout natural mosquito populations [42]. Up until now, there have been no programs or studies about releasing these mosquitoes carrying their symbionts into the environment; only preliminary laboratory studies have been conducted.

*Wolbachia*, a maternally transmitted Gram-negative endosymbiotic bacterium, is a promising tool against diseases transmitted by mosquitoes. *Wolbachia* can be found worldwide in numerous arthropod species. More than 65% of all insect species are naturally infected by it due to its capacity to manipulate cellular and reproductive processes in invertebrates. *Wolbachia* behaves more like a reproductive parasite by inducing feminization of genetic males, parthenogenesis, male-killing and cytoplasmic incompatibility (CI). Although the effects of *Wolbachia* infection on vector reproduction are understood on some level, the exact molecular mechanism of cytoplasmic incompatibility remains unknown despite a substantial amount of research [43]. CI is a conditional embryonic lethality that occurs when males infected with CI-inducing *Wolbachia* strains are crossed with uninfected females (unidirectional CI) or with females carrying other incompatible *Wolbachia* strains (bidirectional CI). The reciprocal mating of uninfected males mated with infected females produces viable *Wolbachia*-infected offspring. CI increases the frequency of *Wolbachia* infection in a given population with each subsequent generation. Uninfected males can be unknowingly contaminated by mating with infected females [44,45,46,47]. Strategies to use this bacterium as a vector control tool include (1) using *Wolbachia*-induced cytoplasmic incompatibility as a form of sterility for a mass male-release strategy analogous to a sterile insect technique and (2) using the reproductive advantage afforded by *Wolbachia*-induced cytoplasmic incompatibility as a population replacement strategy to drive wanted phenotypes into natural populations. These strategies are ways to suppress or modify insect populations to aid in the control of insect pests and disease vectors [43].

The Incompatible Insect Technique (IIT), based on the Sterile Insect Technique (SIT) [48], uses the cytoplasmic incompatibility characteristics of *Wolbachia* to achieve population suppression. Although population replacement approaches can be performed with the simultaneous release of both males and females, population-suppression strategies should aim to release only males. However, both SIT and IIT require a highly efficient sex separation method to avoid accidental release of females, which, in the case of IIT, may result in population replacement of the target population; the females are also potential carriers of pathogens. In the absence of an adequately accurate sexing system, *Wolbachia* and irradiation can be combined to ensure a successful population-suppression approach. Besides *Ae. aegypti*, other mosquito species such as *Aedes polynesiensis* (South Pacific), *Aedes albopictus* (Italy) and *Culex pipiens quinquefasciatus* (southwestern Indian ocean) are also being tested in different countries to demonstrate the feasibility of using this interaction as a population-suppression technology [49]. The use of *Wolbachia* has already motivated an international initiative to control mosquitoes; in the near future, this initiative may be incorporated into the guidelines of several countries as an official strategy for population suppression.

Studies of *Wolbachia* cytoplasmic incompatibility in mosquitoes infected with dengue virus have revealed that the severity of the host infection decreased using a type of *Wolbachia* originally found in *Drosophila*. This fact implies that the *Wolbachia* strain not only was able to reduce the mosquito life span and promote cytoplasmic incompatibility but was also able to reduce dengue infection [50,51,52]. Possible mechanisms for direct viral inhibition by *Wolbachia* include the production of reactive oxygen species by the bacterium and resource competition, such as cholesterol [53]. The next research step is a disease endpoint trial to test the efficacy of the method for dengue and dengue hemorrhagic fever control, ongoing monitoring in and around the release area to test for persistence, and releases to test the spatial spread of the infection across a populated area [54]. Experiments conducted in Australia have established a mosquito able to carry a specific *Wolbachia* population. After a few generations, some mosquitoes collected from the field exhibited the same dengue virus resistance for transmission as in the laboratory [55].

The use of *Wolbachia* as a biocontrol tool to reduce dengue transmission is currently being tested in several countries (www.eliminatedengue.org). *Wolbachia*-infected mosquitoes are released in large numbers over a period of weeks and eventually replace the wild *Ae. aegypti* population. So far, field trials have already been conducted in four sites in Australia, one in Vietnam, and two in Indonesia; trials in Brazil have been underway since September 2014 using *Ae. aegypti* infected with the *w*Mel *Wolbachia* strain. Laboratory and field assays performed in Brazil showed that the *w*Mel infection had no detrimental fitness effects in Brazilian *Ae. aegypti* mosquito populations and would theoretically be able to successfully invade the mosquito populations in distinct urban landscapes. More information can be found at www.eliminatedengue.com/program [47,56].

Mosquitoes carrying *Wolbachia* and forward infected with the dengue virus are not able to transmit the pathogen. According to Hoffmann *et al.* (2011) [54], no virus particle was found in the salivary glands in infections of the DENV-2 serotype. The disruption of dengue transmission by *Wolbachia* through saliva has been demonstrated to be nearly complete in laboratory assays; the strength of the effect is dependent on the *Wolbachia* strain. The strain variability is presumably due to variations in the densities and tissue distributions of *Wolbachia* [54]. For other arboviruses such as chikungunya, *Wolbachia* plays a limited role in the reduction or transmission of the virus [57]. Only a few strains are able to reduce transmission, and *Wolbachia* is not able to provide 100% protection. In some mosquito species, for example *Culex* sp., *Wolbachia* is critically involved in filaria transmission; increasing the number of species carrying *Wolbachia* might also increase the difficulty of controlling filariasis [58].

This symbiont has shown significant potential to be used as a vector-borne disease control; it is far from being an interesting side note in the arthropod literature. The *Wolbachia*-based technique can be enhanced even if it is used with other strategies such as classic vector control strategy and transgenics [58].

## 4. Genetically Modified Mosquitoes

Genetic strategies for vector control are usually divided into two steps. The first step consists of population suppression, containment or eradication that aims to reduce or even eliminate specific insect species by developing genes that are either (conditionally) lethal or capable of making the insects sterile. The second step involves population transformation or replacement. The aim here is not to eliminate the vector but to create a substitution that will be responsible for introducing an effector gene designed to reduce or block disease transmission into the wild population [59,60].

The modification of natural insect populations to combat diseases and agricultural insect pests has existed for approximately 60 years, and the technique is continuously improving. SIT involves the release of a large number of radiation-sterilized males into a target population. When these males mate with native females a decrease in the females’ reproductive potential occurs, which results in the elimination or suppression of the target population if males are released in sufficient numbers over a sufficiently long period of time. The reduction in fecundity decreases both arthropod-borne pathogen transmission as well as the insect population [61,62,63,64,65].

Releases of sterile individuals have permitted the successful regional elimination of important pests such as the New World screwworm fly *Cochliomyia hominivorax* in United States, Mexico and Central America, the Mediterranean fruit fly (Medfly) *Ceratitis capitata*, the pink bollworm *Pectinophora gossypiella* in the United States and the codling moth *Cydia pomonella* in Canada. SIT has also been used to eliminate the tsetse fly (*Glossina fuscipes*), which is a vector of trypanosomiasis, from Zanzibar. SIT is a proven, cost-effective strategy for eradicating or suppressing target populations and protecting areas against invasion or re-invasion [64,65].

SIT was been tested for mosquito control in the 1970s using various sterilizing approaches such as chemosterilization, ionizing radiation, cytoplasmic incompatibility and chromosome translocations [66,67].

Classical SIT trials used DNA-damaging agents such as γ-radiation that generates random dominant lethal mutations in the affected gametes. However, several different approaches have produced modified lines of mosquitoes that can generate sterile males. One example is the release of insects carrying a dominant lethal genetic system (RIDL) that uses a lethal gene expressed in the zygote, rather than in the father, which affords much greater flexibility since the lethal gene can be designed to act at a chosen point during development [68]. In the RIDL system, the offspring resulting from the mating of a transgenic male and a wild female die in the absence of an antibiotic antidote. This method is based on the SIT and also involves the release of a large number of male mosquitoes to compete with wild-type males for a chance to mate with wild females and thus effect population reduction through reduced production of offspring [69,70].

In this review, SIT will refer to male mosquito sterilization resulting from ionizing radiation or genetic modification. Irradiated or genetically modified mosquitoes (GMMs) are currently employed to combat diseases through mosquito vector population-suppression or replacement strategies [60,66,72,73,74].

**Figure 4:**
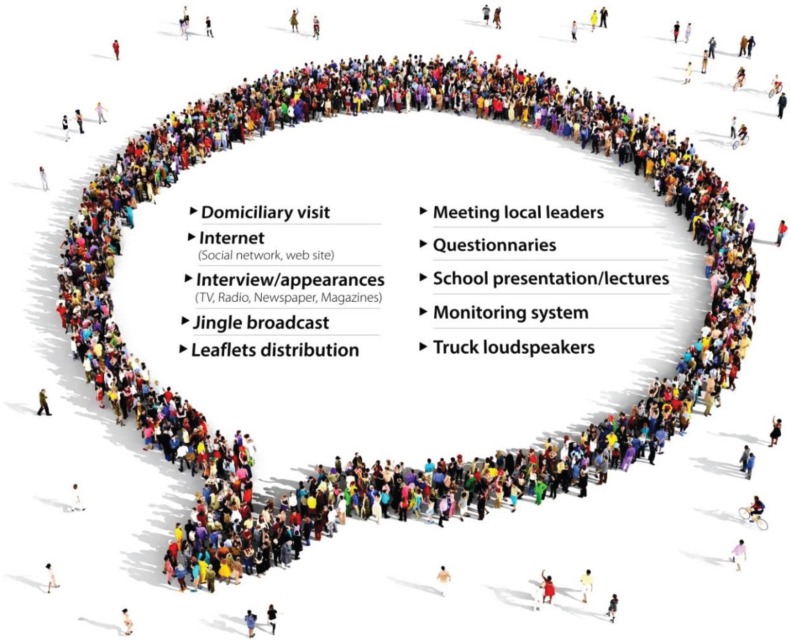
Public engagement activities realized in Bahia, Brazil [82].

Experimental releases of genetically modified (GM) insects have been reportedly occurring in various countries including France, Guatemala, India, Mexico, Panama, Philippines, Singapore, Thailand, the United States and Vietnam [75]. The first open-field trial involving a strain of engineered sterile male *Ae. aegypti* for population suppression took place in the Grand Cayman in 2009 and 2010. The transgenic line caused an approximately 80% suppression of the wild *Ae. aegypti* population in the test area compared with the experimental control area [76,77,78]. GM *Ae. aegypti* mosquitoes were also released in field trials in Malaysia in 2010 [79] and in Brazil in 2011 (manuscript in preparation) [80,81,82]. Results presented at the Regional Meeting of the Integrated Management Strategy for Dengue Prevention and Control held in Panama in 2013 proclaimed that the Brazilian GMM release yielded an 85% reduction in the *Ae. aegypti* population density in areas located in the Juazeiro and Jacobina municipalities (Bahia State) [83]. This project also involved a series of community engagement activities to ensure public awareness about the use of transgenic mosquitoes in integrated pest management (Figure 4) [82].

Methods utilizing SIT to suppress wild mosquito populations have received increased attention in recent years as part of area-wide integrated pest management programs [71,84,85]. The field releases of GMMs are an innovative and now feasible approach to reduce the transmission of many vector-borne diseases [86,87]. Male mosquitoes, unlike female mosquitoes, are not blood feeders; therefore, there is no associated risk of increased biting annoyance or disease transmission. As a result, males are adequate tools for mosquito control [71,88]. Several technologies have been developed to improve the quality of the released sterile insects. In the case of mosquitoes, only males should be released. Therefore, the development of sex separation technologies during mosquito mass rearing is essential.

The International Atomic Energy Agency (IAEA) is funding a Coordinated Research Project (CRP) entitled “Exploring Genetic, Molecular, Mechanical and Behavioural Methods of Sex Separation in Mosquitoes,” which includes researchers from multiple countries. This project aims to develop tools for mosquito SIT such as sexing techniques. The main research axes addressed by the CRP include the development of a genetic sexing strain based on irradiation, classical genetics, molecular genetics and sex separation using mechanical, behavioral and developmental tools [85].

Benedict and Robinson (2003) [66] state that “*Vector control is one of the few proven ways to reduce transmission of many vector-borne diseases. The effect on transmission is identical, regardless of whether the reduction is achieved by artificial means, such as residual spraying with DDT and cultural control, or by natural means, such as unfavorable seasonal climatic conditions or long-term climate change. The success of SIT can build directly upon these demonstrably effective measures*.” [66].

Modeling studies suggest that the use of combined strategies using transgenic mosquitoes (for substituting and/or suppressing mosquito population) and other strategies, *i.e*., *Wolbachia*, can be more effective in terms of vector and disease control than any one strategy used by itself [60,89]. The release of sterile mosquitoes, combined with current control methods, can reduce the mosquito population density by decreasing the probability of contact between a host and a vector, which results in the suppression of disease transmission [71,90,91].

Insecticide control is most effective when applied to high population densities; however, the efficiency of the control decreases rapidly when populations decline and the local population is not completely eliminated. In contrast, SIT becomes more efficient as the population decreases since insects actively seek out areas inaccessible to insecticides [92]. Therefore, insecticide application combined with the release of GMMs can eliminate local populations of vector mosquitoes much more efficiently than the application of insecticides alone, which selects for a resistant population, side effects and pollution.

In Brazil, genetically modified organisms (GMOs) are governed by a Biosafety Law (Law No. 11.105 from March 2005) that lays out inspection and safety rules regulating activities that involve GMOs and creates the National Biosafety Technical Commission (CTNBio). The CTNBio is in charge of assessing direct biologic risks resulting from releasing a GMO into the environment. On 10 April 2014, the CTNBio approved the commercial use of the strain *Ae. aegypti* OX513A developed by Oxitec (Oxfford, UK) through Technical Report 3964/2014. This decision did not focus on issues of technology efficacy, costs or advantages/disadvantages as compared with other technologies of *Ae. aegypti* population control. The CTNBio reports that *Ae. aegypti* pose no additional risks to the environment, human beings and animals [93,94].

Recently, the Florida Keys Mosquito Control District (United States) showed an interest in releasing GMMs in Key Haven (a community on an island adjacent to Key West) as a response to the Key West dengue outbreak of 2009 and 2010. This outbreak was the first dengue episode to occur in the continental United States (not including the Texas-Mexico border) since 1945 and the first locally acquired case in Florida since 1934. Local authorities considered conducting the first United States release of GM male *Ae. aegypti* mosquitoes to prevent dengue dissemination. The Food and Drug Administration Center for Veterinary Medicine (FDA-CVM), the Centers for Disease Control and Prevention (CDC) and the Environmental Protection Agency (EPA) are working together to develop federal regulation about such releases [95,96,97].

According to Oliva *et al.* (2014) [71], the success of control strategies that integrate the liberation of sterile males as part of wider pest control or health-management programs strongly depends on gaining public understanding and acceptance. The integration of SIT should be conducted carefully and requires proper long-term planning. Potential sites for implementing mosquito control programs using SIT are diverse and culturally distinct and require well-adapted ways of disseminating information and public interaction strategies [71].

Brazil’s PNCD should have its current strategies reassessed; the dengue surveillance system should be improved. With the end of open-field trials, the release of transgenic mosquitoes may rapidly become a reality in Brazil. This new technology may confer more effectiveness to Brazil’s vector control planning. Integrated vector management should incorporate as many procedures as possible to ensure long-term success.

## 5. Conclusions

An integrated approach is the most effective strategy to overcome the constant threat of dengue in tropical and subtropical areas in which the existence of main vectors and viral circulation are an undesirable reality. To ensure effective dengue control measures it is necessary to maintain educational and control programs that are governmental priorities. Furthermore, problems such as unplanned urbanization, inadequate water supplies and poor sanitary conditions should be considered and addressed when looking for long-term interventions. Moreover, it is important to note the dengue vaccine development scenario since chemotherapy for this disease is not currently available. An ideal dengue vaccine needs to produce heterotypic immunization; in other words, the seroconversion needs to be efficient for all four serotypes. If not, an immunization may morph into an antibody-dependent enhancement after subsequent infections, a phenomenon inherent in dengue immunopathology [98].

Nowadays, multiple dengue vaccines are in developmental stages. For example, Sanofi-Pasteur and TetraVax-DV dengue vaccines are in the advanced stages in Phase 3 trials, and the preliminary seroconversion results are encouraging but not optimal [99,100]. Nevertheless, some mathematical models suggest that the use of suboptimal vaccines can be beneficial in some epidemic situations [101]. However, even if commercial dengue vaccines are available soon after a successful licensure process, vector control is critical to disrupting the epidemiologic triad of dengue and other emergent/resurgent mosquito-borne viruses that *Ae. aegypti* can also transmit. Thus, an integrated approach focusing on the mosquito vector cannot be disputed.

Therefore, recycling the principles and executive actions focused on the rapid incorporation of new vector control technologies can facilitate essential adaptations to the drastic epidemic scenarios occurring frequently in endemic countries. The goal of this review was to promote a central discussion in relation to the evolution of the national programs supporting dengue control using the case of Brazil as a real-life example. Brazil is taking the first steps to aggregate recent developed technologies such as transgenic mosquitoes and *Wolbachia*. At the same time, the experiences made after trials or the implementation of these technologies in other countries can support strategy modifications as necessary in national programs. However, it is always critical to take into account local characteristics and specific problems.

Research related to vector-borne diseases is currently fertile for innovation. New control methods are arising and showing significant potential for success supported by basic science. Maintaining basic research investments and development incentives can provide a range of tools to increase versatility to deal with different dengue situations. However, regular incorporation of the new methods will strengthen the management capacities of both centralized and decentralized government programs. Prioritizing this view is urgent because the *Ae. aegypti* vector has shown that it is extremely adaptable to human environments and possesses a high reproductive capacity and genetic flexibility, factors that fatally dismantle the best control programs around the world.
